# Impaired Blood Neutrophil Function in the Frequent Exacerbator of Chronic Obstructive Pulmonary Disease: A Proof-of-Concept Study

**DOI:** 10.1007/s00408-016-9930-z

**Published:** 2016-08-16

**Authors:** Arwel Wyn Jones, Richard Robinson, Peer Mohamed, Glen Davison, Hassan Jaysen Izzat, Keir Edward Lewis

**Affiliations:** 1 Lincoln Institute for Health, University of Lincoln, Brayford Pool, Lincoln, LN6 7TS UK; 2 Department of Respiratory Medicine, Prince Philip Hospital, Hywel Dda University Health Board, Llanelli, SA14 8QF UK; 3 School of Sport and Exercise Sciences, University of Kent, Medway Campus, Chatham Maritime, ME4 4AG UK; 4 College of Medicine, Swansea University, Swansea, SA2 8PP UK

**Keywords:** Chronic obstructive pulmonary disease, Elastase, Granulocyte, Polymorphonuclear leukocytes, Reactive oxygen species

## Abstract

**Purpose:**

The underlying biological mechanisms of the frequent exacerbator phenotype of COPD remain unclear. We compared systemic neutrophil function in COPD patients with or without frequent exacerbations.

**Methods:**

Whole blood from COPD frequent exacerbators (defined as ≥2 moderate–severe exacerbations in the previous 2 years) and non-exacerbators (no exacerbations in the preceding 2 years) was assayed for neutrophil function. Neutrophil function in healthy ex-smoking volunteers was also measured as a control (reference) group.

**Results:**

A total of 52 subjects were included in this study: 26 frequent exacerbators, 18 non-exacerbators and 8 healthy controls. COPD frequent exacerbators had blunted blood neutrophil fMLP-stimulated oxidative burst compared to both non-exacerbators (*p* < 0.01) and healthy controls (*p* < 0.001). There were no differences between COPD frequent exacerbators and non-exacerbators in blood neutrophil PMA-stimulated oxidative burst, but both COPD groups had reduced responses compared to healthy controls (*p* < 0.001). Bacterial-stimulated neutrophil degranulation was greater in frequent exacerbators than non-exacerbators (*p* < 0.05).

**Conclusion:**

This study is the first to report aberrant receptor-mediated blood neutrophil function in the frequent exacerbator of COPD.

## Introduction

Chronic obstructive pulmonary disease (COPD) is characterised by a progressive decline in lung function and associated with chronic aberrant inflammatory responses of the lung and airways to noxious stimuli [1]. Globally, COPD is now the third leading cause of mortality [2] and the second leading cause of disability-adjusted life-years lost [3]. Acute exacerbations (defined as sustained worsening of symptoms beyond the normal day-to-day variation that may result in a change of medical treatment and/or hospitalisation) represent one of the primary manifestations of COPD and account for 50–75 % of the costs associated with disease [4]. More frequent exacerbations increase the risk of hospitalisation, contribute to increased mortality risk during hospitalisation and are associated with faster decline in lung function and worsening health-related quality of life [1, 5, 6].

The Evaluation of COPD Longitudinally to Identify Predictive Surrogate Endpoints (ECLIPSE) study proposed that frequent exacerbators are a distinct phenotype in the moderate–severe stages of the disease that is relatively stable over time [7, 8]. More specific phenotyping of COPD appears an increasingly important step towards improving clinical management [8]. Despite clearer recognition of the frequent exacerbator phenotype, the underlying biology of the susceptibility to exacerbations remains unclear [9]. In order to better understand the pathogenesis of exacerbations, comparisons of patients with no versus frequent exacerbations are required [10]. As a result of the identification of large alterations in immune-related gene expression within the blood of frequent exacerbators [11], researching the pathophysiology of COPD exacerbations by focusing on changes that occur in the systemic immune compartment rather than a specific pulmonary immune defect per se could be fruitful.

Neutrophils, particularly neutrophil-derived proteinases, have been implicated in lung destruction and remodelling and hence pathogenesis of COPD [12]. Neutrophil count and neutrophilic inflammatory mediators are higher within the airways, even in the stable (non-exacerbated) state in COPD [13]. Blood neutrophils also show an altered pattern of activity in the stable state in COPD, with impaired chemotaxis and reduced intracellular reactive oxygen species (ROS) production [14–16]. Evidence of further systemic immune dysregulation occurs during exacerbations with circulating neutrophils displaying up-regulation of inflammation-related genes, enhanced expression of cell adhesion molecules and elevated production of elastase and ROS [17–20]. Despite an abundance of data supporting the hypothesis that neutrophils are key effector cells in the development and progression of COPD [13], we could find no studies assessing blood neutrophil function of COPD patients according to exacerbation history.

To better establish the biological underpinning of the frequent exacerbator and ultimately direct development of novel therapies for this high-risk group of patients, we need an improved understanding of the changes in systemic immune function associated with this phenotype. With this in mind, this proof-of-concept study was designed to characterise blood neutrophil function of COPD patients in a stable state with or without a history of frequent exacerbations. The primary aim of this study was to test the null hypothesis that there is no difference in in vitro blood neutrophil function between COPD frequent exacerbators, COPD non-exacerbators and healthy controls.

## Methods

### Study Design and Participants

Following loco-regional ethics approval, 44 patients with COPD, defined as over 40 years, at least 10-pack-year smoking history and post-bronchodilator (100 mcg of inhaled salbutamol) FEV1 <80 % predicted with FEV1/FVC ratio <0.70 [1], were prospectively recruited from outpatient clinics of a UK district hospital.

Current symptoms (cough, sputum), exacerbation history, co-morbidities, prescribed medications and smoking history were collected at interview. Participants underwent clinical examination and spirometry (Vitalograph Alpha^®^, Vitalograph Ltd., UK). Frequent exacerbators were patients who had 2 or more exacerbations requiring oral corticosteroids and/or antibiotics during the last 2 years, and/or attendance to hospital [1]. Patients were deemed non-exacerbators if they had not attended hospital nor required systemic treatments for their COPD during the previous 2 years. Self-reports were confirmed through hospital records and GP prescriptions. Eight healthy, ex-smokers who had no symptoms of lung disease and had normal spirometry were recruited as a control (reference) group. Informed consent was obtained from all individual participants included in the study.

We excluded current smokers defined as anyone reporting smoking a cigarette within 6 months or having an exhaled carbon monoxide (eCO) >10 parts per million (MicroCO, CareFusion Ltd., UK) on the day of testing. We also excluded anyone with known structural lung disease (asthma, bronchiectasis, pulmonary fibrosis); cancer (other than non-melanotic skin cancer); severe renal failure (calculated eGFR less than 60 ml/min) or liver failure; immunodeficiency or autoimmune conditions; anyone prescribed long-term antibiotics (including azithromycin), aminophylline, maintenance oral steroids or other immunosuppressive medications. We included patients prescribed inhaled corticosteroids (ICS), anticholinergics and long- and short-acting beta agonists. All patients were prescribed optimal medication for their COPD according to current guidelines [1] and all were deemed clinically stable with none reporting a worsening of symptoms (no exacerbation) in the previous 3 months.

### Sample Collection and Haematological Analysis

Participants provided 10 ml of blood (K_3_EDTA and lithium heparin) from the antecubital vein. Total and differential leukocyte counts and platelets were recorded on K_3_EDTA anticoagulated whole blood using an automated haematology analyser (ADVIA 2120, Siemens Healthcare Diagnostics GmbH, Eschborn, Germany).

### Neutrophil Assays

As described previously [21], neutrophil phorbol-12-myristate-13-acetate (PMA)- and formyl-methionyl-leucyl-phenylalanine(fMLP)-stimulated oxidative burst were assessed by a chemiluminescence (CL) kit (ABEL^®^04 M, Knight Scientific Ltd, Plymouth, UK) incorporating the light-emitting protein Pholasin^®^. The CL per well was measured by a microplate luminometer (FLUOstar OPTIMA, BMG Labtech, Aylesbury, UK). Each well contained 10 µL of diluted whole (K_3_EDTA) blood (ratio of 1:100 with Hank’s balanced salt solution; HBSS, without calcium and magnesium), 90 µL assay buffer (HBSS with calcium and magnesium), 50 µL Pholasin and 20 µL adjuvant K. These mixtures were gently shaken and incubated at 37 °C for 30 s in the luminometer, prior to the addition of 20 µL of PMA (5 µg/mL), 20 µL fMLP (10 µM) (or additional 20 µL of HBSS for unstimulated wells) to provide an end total volume of 200 µL per well, a 1:1010 final blood dilution and stimulated wells containing a PMA or fMLP concentration of 0.5 µg/mL or 1 µM. These concentrations have been standardised to be not rate limiting, even in the presence of abnormally high numbers of leukocytes, and thus provide responses that are reproducible. For PMA, CL of stimulated and unstimulated replicates of the same samples was recorded as relative light units (RLU) at 20-s intervals for 30 min. For fMLP, CL was recorded every second for 300 s. The area under the unstimulated CL curves was subtracted from stimulated curves of the same sample to determine PMA- or fMLP-stimulated oxidative burst. To calculate responses on a per-cell basis in whole blood, area under the CL curve was expressed as the number of neutrophils (in well) only as the contributions of monocytes, eosinophils and basophils in our 1:1010 final dilution of blood are considered to be insignificant [21, 22].

Neutrophil-stimulated degranulation was determined as previously described [23]. Heparinised blood (1 mL) was added to a microcentrifuge tube with 50 µL bacterial stimulant containing *Staphylococcus aureus, Pseudomonas fluorescens* and *Aerobacter aerogenes* (840-15, Sigma, Poole, UK). The microcentrifuge tubes were initially mixed by gentle inversion before incubation at 37 °C for 1 h (all tubes also mixed halfway through). Following incubation, the tubes were centrifuged for 2 min at 16,000 *g*, with the supernatant being immediately removed and stored at −80 °C until further analysis. Following thawing at room temperature, α1-proteinase inhibitor (α1-PI)/neutrophil elastase (NE) complex was measured in all samples using an ELISA kit (Calbiochem^®^, Merck, Darmstadt, Germany). Bacterial-stimulated degranulation was based on subtracting α1-PI/NE of unstimulated samples (heparinised plasma at the same time point) away from the stimulated samples and expression per neutrophil.

### Statistical Analysis

Statistical analysis was performed using SPSS (v21.00; SPSS Inc., Chicago, IL, USA). Normality was tested using the Shapiro–Wilk test, and statistical significance was set at *p* < 0.05. Primary outcome measures were stimulated neutrophil function. Data were analysed between groups using one-way analysis of variance and independent *t* tests or Kruskal–Wallis and Mann–Whitney *U* tests. Relationship between baseline neutrophil count and daily beclomethasone dose with neutrophil function were assessed using Pearson correlation. For categorical data (gender), Fisher’s exact test was applied.

## R**esults**

### Clinical Characteristics and Blood Leukocytes

Clinical details and total and differential leukocyte counts are summarised in Table 1. As expected, FEV1 was significantly greater in healthy controls compared to both COPD groups (*p* < 0.001). No significant differences were found between COPD frequent exacerbators and non-exacerbators in FEV1, prescribed daily beclomethasone equivalent dose and leukocyte counts (*p* > 0.05).

**Table 1:** Clinical characteristics of study participants

Parameter	Healthy control (*n* = 8)	COPD non-exacerbator (*n* = 18)	COPD frequent exacerbator (*n* = 26)	*p* value
Age (years)	63.3 ± 7.6	68.7 ± 7.7	65.0 ± 7.5	0.15
Males/females (*n*)	6/2	11/7	17/9	0.80
FEV_1_ (L)	2.7 ± 0.4	1.0 ± 0.3	1.0 ± 0.5	<0.01
FEV_1_ (% predicted)	89.7 ± 8.5	38.5 ± 10.8	36.6 ± 13.4	<0.01
Daily beclomethasone equivalent (µg)		800 (0, 2000)	1500 (950, 2000)	0.14
Total leukocytes (10^9^ L^−1^)	7.0 (6.3, 7.7)	9.1 (6.9, 10.7)	7.1 (6.2, 8.9)	0.13
Neutrophils (10^9^ L^−1^)	4.0 (3.7, 4.7)	6.0 (4.3, 7.4)	4.6 (4.0, 6.3)	0.05
Monocytes (10^9^ L^−1^)	0.5 (0.4, 0.7)	0.5 (0.4, 0.6)	0.5 (0.3, 0.8)	0.70
Total lymphocytes (10^9^ L^−1^)	1.7 (1.6, 2.1)	1.7 (1.2, 2.2)	1.6 (1.0, 2.1)	0.51
Neutrophil:lymphocyte ratio	2.3 (1.8, 2.9)	3.6 (2.6, 5.1)	3.2 (2.0, 4.8)	0.06
Platelets (10^9^ L^−1^)	236 (221, 333)	284 (246, 389)	289 (238, 380)	0.53

Data are presented as mean ± standard deviation or median (interquartile range). *FEV1* forced expiratory volume in 1 s

### Neutrophil Oxidative Burst

**Fig. 1:**
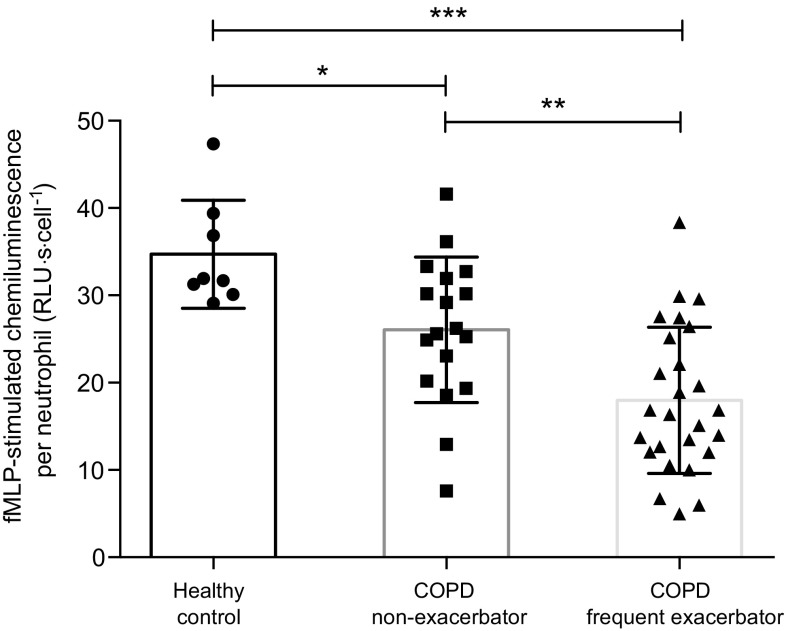
Neutrophil fMLP-stimulated oxidative burst (chemiluminescence) responses. *Columns* indicate mean values for each group. *Error bars* represent standard deviation. Significant difference between groups: *p < 0.05, **p < 0.01, ***p < 0.001

Both COPD frequent exacerbators and non-exacerbators had lower fMLP-stimulated oxidative neutrophil burst compared to controls, with frequent exacerbators also showing significantly lower function compared to non-exacerbators (Fig. 1).

**Fig. 2:**
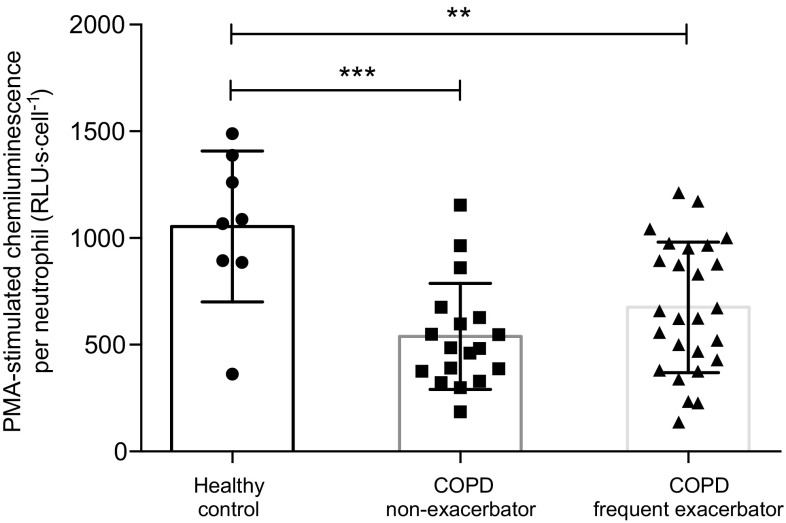
Neutrophil PMA-stimulated oxidative burst (chemiluminescence) responses. *Columns* indicate mean values for each group. *Error bars* represent standard deviation. Significant difference between groups: **p < 0.01, ***p < 0.001

Neutrophil PMA-stimulated oxidative burst was significantly lower in both COPD groups compared to controls (Fig. 2). However, there was no significant difference between COPD frequent exacerbators and non-exacerbators for responses to PMA (*p* = 0.45). To help determine the effect of baseline inflammatory status on subsequent neutrophil responsiveness, we investigated whether ICS exposure or baseline blood neutrophil count correlated with measures of neutrophil function. There were no correlations between fMLP-stimulated response and baseline neutrophil count (*p* = 0.42), or between fMLP-stimulated response and daily beclomethasone equivalent dose (*p* = 0.17). There were no correlations between PMA-stimulated oxidative burst and baseline neutrophil count (*p* = 0.791) or between PMA-stimulated responses and daily beclomethasone equivalent dose (*p* = 0.30).

### Neutrophil Degranulation

**Fig. 3:**
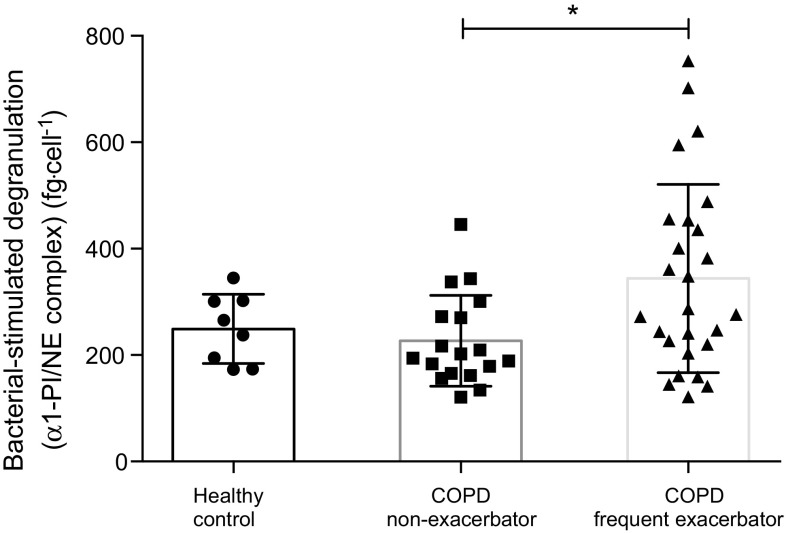
Bacterial-stimulated neutrophil degranulation (α1-proteinase inhibitor/neutrophil elastase). *Columns* indicate mean values for each group. *Error bars* represent standard deviation. Significant difference between groups: *p < 0.05

Blood neutrophils of COPD frequent exacerbators showed heightened bacterial-stimulated degranulation (α1-PI/NE complex) compared to non-exacerbators (Fig. 3). Although there was no correlation between baseline neutrophil count and stimulated concentrations of α1-PI/NE complex (*p* = 0.880), there was a significant positive correlation between daily beclomethasone equivalent dose and bacterial-stimulated degranulation (*p* = 0.04, *r* = 0.298).

## Discussion

In the present study, we showed that blood neutrophil oxidative burst is blunted in COPD with receptor-dependent ROS production showing greater impairment in the frequent exacerbator phenotype. Assessment of total degranulation responses of blood neutrophils showed further dysregulation associated with the exacerbator phenotype.

To the best of our knowledge, this is the first report on the distinct patterns of neutrophil function that relate to COPD exacerbation phenotype whilst in a stable state. We do not prove mechanistically that changes in neutrophil effector functions are the causes of or the results of frequent exacerbations but provide an important starting point for future investigations.

This study supports previously reported evidence of reduced intracellular oxidative burst to fMLP in COPD populations compared to healthy counterparts [15, 24]. Impaired chemotactic responses to fMLP have also been observed in moderate–severe COPD, compared to healthy smokers and non-smokers as well as COPD patients with milder airflow obstruction [16]. These impaired functional responses to stimulants are consistent with poor resistance to infection in COPD. Like us, some other studies suggest that greater severity of disease is not related with augmented activity of inflammatory cells but a down-regulation. Our study, however, suggests that there are certain clinical phenotypes, which show further changes in neutrophil responses to inflammatory stimuli (bacterial peptides) that may partly explain their intrinsic susceptibility to recurrent infectious episodes. Blood neutrophils in COPD demonstrate reduced migratory accuracy towards fMLP and decreased structural changes and sensitivity to such chemotactic factors under receptor occupancy [14]. Aberrant blood neutrophil responses in COPD appear to be due to intrinsic cell defect (e.g. intracellular enzymatic reactions and kinases) rather than cell surface expression of chemoattractant receptors [14]. Further investigation (utilising whole blood flow cytometry) of immune regulation events upstream (e.g. at the level of FPR1 receptor) and downstream of neutrophil activation would help understand the interpretation and significance of impaired fMLP-stimulated oxidative burst in the frequent exacerbator.

Our findings of reduced blood neutrophil PMA oxidative burst in COPD contrast with earlier reports of greater ROS production compared to controls [25, 26]. Differences in results could be explained by study participants (including the differences in treatment (e.g. non-ICS users in [25, 26]) or characteristics of the sampling and assays. For example, Renkema et al. [25] used heparinised samples (as opposed to EDTA), which have been demonstrated to interfere with neutrophils prior to subsequent activation of oxidative burst [27, 28]. Previous studies [25, 26] had also used isolation procedures that are known to influence neutrophil activation (including density gradient centrifugation, fluctuations in temperature) prior to any staining and in vitro stimulation. Our approach, using whole blood, provides minimal manipulation of cells and provides a more accurate representation of neutrophil behaviour in vivo (i.e. better maintenance of the extracellular milieu) [29]. In contrast to fMLP, we did not observe differences between exacerbation phenotypes in PMA-stimulated oxidative burst. Unlike the G-coupled receptor-dependent responses to fMLP, PMA penetrates the cell (independent of a receptor), triggering a long-lasting, strong stimulation via protein kinase C and activation of NADPH oxidase throughout the cell. PMA is considered an artificial stimulus (not encountered in vivo), differing substantially from physiological agonists (e.g. fMLP) [30]. Although PMA-stimulated responses provides further evidence of the aberrant intracellular signalling in COPD, such cell activation lacked biological sensitivity to characterise COPD phenotypes that may differ in their ability to recognise microbial moieties (e.g. formylated peptides) and mount responses toward infectious/inflammatory challenge [31].

COPD blood neutrophils possess exaggerated innate immune responses to Toll-like receptor (TLR) agonists (e.g. lipopolysaccharide) [32]. Upon triggering neutrophil degranulation, the concentration of free NE increases for only a brief period of time as its major inhibitors (e.g. α1-PI) rapidly reach equimolar concentrations [33]. Measurement of α1-PI/NE complexes is considered to be a marker of total NE release during neutrophil degranulation [34]. Here, we suggest that such heightened responses to TLR agonists (in our case both gram-positive and gram-negative bacteria) are more reflective of a COPD frequent exacerbator. These findings appear contradictory to the data on fMLP, whereby both fMLP-stimulated oxidative burst (e.g. FRP1) and bacterial-stimulated degranulation (e.g. TLR2, TLR4) represent receptor-mediated events. Similar differences between circulating phagocyte responses to chemoattractants and pro-inflammatory mediators have been observed in COPD [35]. Although distinct and complex intracellular transduction pathways are involved, one plausible mechanism for the observed variability between neutrophil effector functions in our study is the selective effect of ICS on one of the neutrophil function pathways (α1-PI/NE). In COPD patients stratified according to GOLD severity, increasing ICS dosage was associated with enhanced stimulated neutrophil degranulation [36]. Budesonide and fluticasone propionate prolong human neutrophil survival by inhibiting apoptosis at clinically relevant drug concentrations [37]. We speculate that the greater (albeit non-significant) mean beclomethasone exposure of the frequent exacerbators may partly explain the heightened NE release of stimulated blood neutrophils in the frequent exacerbator.

Strengths of our study include its real-life setting using recognised clinical phenotypes of COPD. Our findings are generalisable with patient demographics reflecting patients with moderate-to-severe disease that attend a standard secondary care service. The greater proportion of patients prescribed ICS in frequent exacerbators corroborates previous findings [11] and reflects current treatment guidelines [1]. The number of participants in our study is comparable to most other biological studies comparing neutrophil responses in COPD. We also validated and controlled for smoking status and used a healthy control group as an additional reference point.

The cross-sectional methodology does not allow us to identify whether responses of the frequent exacerbator represent an intrinsic or acquired defect of neutrophil function. The primary aim of this study was to characterise blood neutrophil function (in the stable state) in COPD exacerbation phenotypes and not the temporal nature of the relationship between inflammatory mediators and onset of COPD exacerbations investigated previously [38]. We did not sample airway neutrophils to compare against blood; however, others propose that the dysregulation of immune function in COPD frequent exacerbators may be systemic rather than a specific abnormality limited to the lungs [12].

## Conclusions

In conclusion, we have demonstrated aberrant blood neutrophil functions in COPD, highlighting alterations in receptor-dependent responses that relate to the frequent exacerbator phenotype. Frequent exacerbators have impaired oxidative responses to chemotactic factors and augmented degranulation responses to bacterial triggers in the circulation. Importantly, this study provides further support to a biological underpinning of the frequent exacerbator phenotype and provides insights into immune cell defects that can act as the basis for future investigations.

## Abbreviations

α1-PI: Alpha 1-proteinase inhibitor
CL: Chemiluminescence
eCO: Exhaled carbon monoxide
fMLP: Formyl-methionyl-leucyl-phenylalanine
HBSS: Hank’s balanced salt solution
NADPH: Nicotinamide adenine dinucleotide phosphate
NE: Neutrophil elastase
PMA: Phorbol-12-myristate-13-acetate
ROS: Reactive oxygen species
RLU: Relative light units
TLR: Toll-like receptor

## References

[1] Global initiative for chronic obstructive lung disease. Global strategy for diagnosis, management, and prevention of COPD 2016. http://www.goldcopd.org/. Accessed 28 Jan 2016

[2] Lozano R, Naghavi M, Foreman K (2012). Global and regional mortality from 235 causes of death for 20 age groups in 1990 and 2010: a systematic analysis for the Global Burden of Disease Study 2010. Lancet.

[3] Murray CJ, Lopez AD (2013). Measuring the global burden of disease. N Engl J Med.

[4] Celli BR, MacNee W, ATS/ERS committee members (2004). Standards for the diagnosis and treatment of patients with COPD: a summary of the ATS/ERS position paper. Eur Respir J.

[5] Donaldson GC, Wilkinson TM, Hurst JR (2005). Exacerbations and time spent outdoors in chronic obstructive pulmonary disease. Am J Respir Crit Care Med.

[6] Seemungal TA, Donaldson GC, Paul EA (1998). Effect of exacerbation on quality of life in patients with chronic obstructive pulmonary disease. Am J Respir Crit Care Med.

[7] Hurst JR, Vestbo J, Anzueto A (2010). Evaluation of COPD Longitudinally to Identify Predictive Surrogate Endpoints (ECLIPSE) Investigators. Susceptibility to exacerbation in chronic obstructive pulmonary disease. New Engl J Med.

[8] Vestbo J, Agusti A, Wouters EFM (2014). Evaluation of COPD Longitudinally to Identify Predictive Surrogate Endpoints (ECLIPSE) Investigators. Should we view chronic obstructive pulmonary disease differently after ECLIPSE? A clinical perspective from the study team. Am J Respir Crit Care Med.

[9] Berenson CS, Kruzel RL, Eberhardt E (2014). Impaired innate immune alveolar macrophage response and the predilection for COPD exacerbations. Thorax.

[10] Agusti A, Vestbo J (2011). Current controversies and future perspectives in chronic obstructive pulmonary disease. Am J Respir Crit Care Med.

[11] Singh D, Fox SM, Tal-Singer R (2014). Altered gene expression in blood and sputum in COPD frequent exacerbators in the ECLIPSE cohort. PLoS ONE.

[12] Sapey E, Stockley RA (2006). COPD exacerbations: aetiology. Thorax.

[13] Stockley JA, Walton GM, Lord JM (2013). Aberrant neutrophil functions in stable chronic obstructive pulmonary disease: the neutrophil as an immunotherapeutic target. Int Immunopharmacol.

[14] Sapey E, Stockley JA, Greenwood H (2011). Behavioral and structural differences in migrating peripheral neutrophils from patients with chronic obstructive pulmonary disease. Am J Respir Crit Care Med.

[15] Wehlin L, Lofdahl M, Lundahl J (2005). Reduced intracellular oxygen radical production in whole blood leukocytes from COPD patients and asymptomatic smokers. Chest.

[16] Yoshikawa T, Dent G, Ward J (2007). Impaired neutrophil chemotaxis in chronic obstructive pulmonary disease. Am J Respir Crit Care Med.

[17] Fujimoto K, Yasuo M, Urushibata K (2005). Airway inflammation during stable and acutely exacerbated chronic obstructive pulmonary disease. Eur Respir J.

[18] Noguera A, Busquets X, Sauleda J (1998). Expression of adhesion molecules and G proteins in circulating neutrophils in chronic obstructive pulmonary disease. Am Respir J Crit Care Med.

[19] Oudijk EJ, Nijhuis EH, Zwank MD (2005). Systemic inflammation in COPD visualised by gene profiling in peripheral blood neutrophils. Thorax.

[20] Vaitkus M, Lavinskiene S, Barkauskiene D (2013). Reactive oxygen species in peripheral blood and sputum neutrophils during bacterial and nonbacterial acute exacerbation of chronic obstructive pulmonary disease. Inflammation.

[21] Jones AW, Thatcher R, March DS (2015). Influence of 4 weeks of bovine colostrum supplementation on neutrophil and mucosal immune responses to prolonged cycling. Scand J Med Sci Sports.

[22] Morozov VI, Pryatkin SA, Kalinski MI (2003). Effect of exercise to exhaustion on myeloperoxidase and lysozyme release from blood neutrophils. Eur J Appl Physiol.

[23] Davison G, Diment BC (2010). Bovine colostrum supplementation attenuates the decrease of salivary lysozyme and enhances the recovery of neutrophil function after prolonged exercise. Br J Nutr.

[24] Fietta A, Bersani C, De Rose V (1988). Evaluation of systemic host defense mechanisms in chronic bronchitis. Respiration.

[25] Renkema TE, Postma DS, Noordhoek JA (1993). Influence of in vivo prednisolone on increased in vitro O_2_-generation by neutrophils in emphysema. Eur Respir J.

[26] Noguera A, Batle S, Miralles C (2001). Enhanced neutrophil response in chronic obstructive pulmonary disease. Thorax.

[27] Freitas M, Porto G, Lima JL (2008). Isolation and activation of human neutrophils in vitro. The importance of the anticoagulant used during blood collection. Clin Biochem.

[28] Kuijpers TW, Tool AT, van der Schoot CE (1991). Membrane surface antigen expression on neutrophils: a reappraisal of the use of surface markers for neutrophil activation. Blood.

[29] van Eeden SF, Klut ME, Walker BA (1999). The use of flow cytometry to measure neutrophil function. J Immunol Methods.

[30] Decoursey TE, Ligeti E (2005). Regulation and termination of NADPH oxidase activity. Cell Mol Life Sci.

[31] Jaillon S, Galdiero MR, Del Prete D (2013). Neutrophils in innate and adaptive immunity. Semin Immunopathol.

[32] Baines KJ, Simpson JL, Gibson PG (2011). Innate immune responses are increased in chronic obstructive pulmonary disease. PLoS One.

[33] Carter RI, Mumford RA, Treonze KM (2011). The fibrinogen cleavage product Aalpha-Val360, a specific marker of neutrophil elastase activity in vivo. Thorax.

[34] Carter RI, Ungurs MJ, Mumford RA (2013). Aalpha-Val360: a marker of neutrophil elastase and COPD disease activity. Eur Respir J.

[35] Aldonyte R, Jansson L, Piitulainen E (2003). Circulating monocytes from healthy individuals and COPD patients. Respir Res.

[36] Vlahos R, Wark PA, Anderson GP (2012). Glucocorticosteroids differentially regulate MMP-9 and neutrophil elastase in COPD. PLoS One.

[37] Zhang X, Moilanen E, Kankaanranta H (2001). Beclomethasone, budesonide and fluticasone propionate inhibit human neutrophil apoptosis. Eur J Pharmacol.

[38] Koutsokera A, Stolz D, Loukides S (2012). Systemic biomarkers in exacerbations of COPD: the evolving clinical challenge. Chest.

